# The Italian registry for patients with Prader–Willi syndrome

**DOI:** 10.1186/s13023-023-02633-5

**Published:** 2023-02-15

**Authors:** Marco Salvatore, Paola Torreri, Graziano Grugni, Adele Rocchetti, Mohamad Maghnie, Giuseppa Patti, Antonino Crinò, Maurizio Elia, Donatella Greco, Corrado Romano, Adriana Franzese, Enza Mozzillo, Annamaria Colao, Gabriella Pugliese, Uberto Pagotto, Valentina Lo Preiato, Emanuela Scarano, Concetta Schiavariello, Gianluca Tornese, Danilo Fintini, Sarah Bocchini, Sara Osimani, Luisa De Sanctis, Michele Sacco, Irene Rutigliano, Maurizio Delvecchio, Maria Felicia Faienza, Malgorzata Wasniewska, Domenico Corica, Stefano Stagi, Laura Guazzarotti, Pietro Maffei, Francesca Dassie, Domenica Taruscio

**Affiliations:** 1 National Centre for Rare Diseases, Undiagnosed Rare Diseases Interdepartmental Unit, Istituto Superiore di Sanità, Viale Regina Elena 299, 00161 Rome, Italy; 2 Division of Auxology, Istituto Auxologico Italiano IRCCS, Piancavallo di Oggebbio, VB Italy; 3 Department of Pediatrics, IRCCS Istituto Giannina Gaslini, Genoa, Italy; 4 Department of Neuroscience, Rehabilitation, Ophthalmology, Genetics, Maternal and Child Health, University of Genova, Genoa, Italy; 5 Center for Rare Diseases and Congenital Defects, Fondazione Policlinico Universitario “Agostino Gemelli” IRCCS, Rome, Italy; 6 Oasi Research Institute – IRCCS, Troina, EN Italy; 7 Department of Biomedical and Biotechnological Sciences, University of Catania, Catania, Italy; 8 AOU Federico II, Naples, Italy; 9 Unità di Endocrinologia e Prevenzione e Cura del Diabete, IRCCS Azienda Ospedaliero-Universitaria di Bologna, Dipartimento di Scienze Mediche e Chirurgiche (DIMEC), Alma Mater Studiorum Università di Bologna, Bologna, Italy; 10 Rare Diseases Unit, Department of Pediatrics, IRCCS AOU Sant’Orsola, Bologna, Italy; 11 IRCCS Materno Infantile Burlo Garofolo, Trieste, Italy; 12 IRCCS Bambino Gesù Paediatric Hospital, Rome, Italy; 13 IRCCS Ospedale San Raffaele, Milan, Italy; 14 AOU Città della Salute e della Scienza/Ospedale Infantile Regina Margherita and Dipartimento di Scienze di Sanità Pubblica e Pediatriche, Università di Torino, Turin, Italy; 15 IRCCS Casa Sollievo della Sofferenza - San Giovanni Rotondo, Foggia, Italy; 16 AOU Consorziale Policlinico Giovanni XXIII, Bari, Italy; 17 AOU Policlinico “G. Martino”, Messina, Italy; 18 Azienda Ospedaliera Universitaria Meyer, Florence, Italy; 19 Azienda Ospedale Università Padova (LG), Padua, Italy; 20 Dipartimento di Medicina (DIMED), University of Padua (PM, FD), Padua, Italy

**Keywords:** Prader–Willi syndrome, Registry, Genetic diseases, Rare diseases, Quality

## Abstract

**Background:**

Prader–Willi syndrome (PWS) is a rare and complex genetic disease, with numerous implications on metabolic, endocrine, neuropsychomotor systems, and with behavioural and intellectual disorders. Rare disease patient registries are important scientific tools (1) to collect clinical and epidemiologic data, (2) to assess the clinical management including the diagnostic delay, (3) to improve patients’ care and (4) to foster research to identify new therapeutic solutions. The European Union has recommended the implementation and use of registries and databases. The main aims of this paper are to describe the process of setting up the Italian PWS register, and to illustrate our preliminary results.

**Materials and methods:**

The Italian PWS registry was established in 2019 with the aims (1) to describe the natural history of the disease, (2) to determine clinical effectiveness of health care services, (3) to measure and monitor quality of care of patients. Information from six different variables are included and collected into this registry: demographics, diagnosis and genetics, patient status, therapy, quality of life and mortality.

**Results:**

A total of 165 patients (50.3% female vs 49.7% male) were included into Italian PWS registry in 2019–2020 period. Average age at genetic diagnosis was 4.6 years; 45.4% of patients was less than 17 years old aged, while the 54.6% was in adult age (> 18 years old). Sixty-one percent of subjects had interstitial deletion of the proximal long arm of paternal chromosome 15, while 36.4% had uniparental maternal disomy for chromosome 15. Three patients presented an imprinting centre defect and one had a de novo translocation involving chromosome 15. A positive methylation test was demonstrated in the remaining 11 individuals but the underlying genetic defect was not identified. Compulsive food-seeking and hyperphagia was present in 63.6% of patients (prevalently in adults); 54.5% of patients developed morbid obesity. Altered glucose metabolism was present in 33.3% of patients. Central hypothyroidism was reported in 20% of patients; 94.7% of children and adolescents and 13.3% of adult patients is undergoing GH treatment.

**Conclusions:**

The analyses of these six variables allowed to highlight important clinical aspects and natural history of PWS useful to inform future actions to be taken by national health care services and health professionals.

## Introduction

Prader–Willi syndrome (PWS) is a rare and complex genetic disease; it is mainly characterized by severe neonatal hypotonia with feeding difficulties in the first months of life, followed by global developmental delays, hyperphagia and gradual development of morbid obesity in later childhood, if uncontrolled [1]. Patients show typical dysmorphic facial features, acromicria, kyphoscoliosis, strabismus, and other musculoskeletal disorders [2]. PWS has numerous implications on metabolic, endocrine, and neuropsychomotor systems and patients may display behaviour abnormalities, mild to moderate intellectual disability and high incidence of psychosis.

PWS has an estimated prevalence of 1/10000 to 1/30000 [2, 3] and an incidence of approximately 1 in 21,000/births [4], resulting the most common syndromic cause of morbid obesity. PWS affects males and females equally, without difference between ethnicities. The mainstay of diagnosis is DNA methylation testing to identify any defect in the parental imprinting within the PWS critical region on chromosome 15, and this test can detect more than 99% of all affected individuals [1, 2, 5]. In developed countries, diagnosis can be made early in life at around 18 days of age [3]. Nonetheless, in most cases, confirmation is done at mean age of 3.9 years, even in centres of reference. Globally, the number of individuals with PWS is approximately 400.000, and 20.000 of these cases are in the United States [6–8].

From a genetic point of view, PWS is an imprinting disorder and results from the loss of expression of a cluster of paternally expressed genes on 15q11.2-q13 chromosome. The most common genetic abnormalities include paternal deletion (60–65%) of cases, maternal uniparental disomy (30–35%) and defects in the imprinting center on chromosome 15, such as microdeletions or epimutations, or paternal chromosomal translocation in the remaining individuals [5]. Of note, some cases with mosaic methylation pattern have been also described [9].

The complexity of this multifaceted syndrome makes its clinical management particularly challenging and highlights the role of the inter-professional clinical team in improving patients’ care [10].

Rare disease registries are important tools to collect and share data, to enable multidisciplinary collaboration with the overall aim of improving patients’ care and to support healthcare management [11]. Since 2008 the European Commission recommended Member States to make collaborative efforts to establish registries and databases on rare disease as key instruments to increase knowledge on these disorders and develop clinical research. In fact, they are the only way to pool data achieving a sufficient sample size for epidemiological research and/or clinical research [12].

Moreover, the European Commission acknowledges the relevance to improve standardization and data comparability among patient registries and promoted the use of common data elements for the collection of comparable data [13].

Finally, rare disease registries are key to Evidence-Based Personalized Medicine [14].

With this background, the Italian PWS registry was launched in 2019 using a standardized and customizable web-based platform named RegistRare, developed by the National Centre for Rare Diseases (NCRD) at the Istituto Superiore di Sanità (ISS). RegistRare is available to collect clinical and epidemiological data on specific rare diseases in Italy after a formal agreement among stakeholders involved in a specific rare disease, such as patients’ associations, clinicians and researchers [15].

The Italian PWS registry is based on the official agreement among the Italian Federation PWS-Onlus, the referral PWS centres within Italian hospitals and the NCRD of the ISS.

Its aims at collecting clinical and epidemiologic data to improve and standardize the management of patients at national level, to reduce morbidity and mortality, to accelerate research and to support the development of treatments for PWS assessing the long-term patient outcomes. Specific aims of the Registry are (1) to study the PWS natural history; (2) to understand the full spectrum of PWS features across the entire population; (3) to identify unmet medical needs, rare complications, and understudied areas; (4) to facilitate partnerships with stakeholders; (5) to expedite completion of clinical trials; (6) to guide the development of standards of care; and (7) to allow participants to centrally store their PWS medical data.

## Materials and methods

### Registry architecture and security

The Italian PWS registry is a web-based database, in compliance with EU criteria and standards [16] and it is hosted on the web-based Platform “RegistRare”. It has been developed as a collaboration among ISS, patient advocacy groups, and clinicians from Italian reference centres involved in PWS. The Registry is compliant with Health Information Privacy Laws, and the security requirements of the European Union General Data Protection Regulation (GDPR). Registry data is only accessed by registry study personnel. De-identified data can be shared with researchers and other stakeholders as per the Registry protocol. All protocols for patient’s inclusion are reviewed and approved by a Scientific Committee (SC).

Additionally, to meet registry objectives and facilitate further eventual sharing with global registries, Findable, Accessible, Interoperable, and Reusable (FAIR) Guiding Principles [17] have been included in the developing of the Italian PWS registry. These principles refer to data being *findable* in discipline specific search engines or directories via data identifiers and metadata; *accessible* via clear, transparent protocols or policies; *interoperable* via the use of community or discipline approved formats and terminologies for data and metadata, as well as linking of metadata to related resources via identifiers and *reusable* by retaining data richness, provision of a machine readable licence and source information, and available metadata to facilitate reuses.

The data are provided based on a dedicated informed consent (IC) signed by each patient and/or by the legal representative, also providing for the possibility of its revocation. All the information relating to the study are provided to subjects in accordance with the rules of good clinical practice and current regulations. The required data are drawn up from the literature and an exhaustive list of potential data (including outcomes of interest). The study protocol is conformed with the Declaration of Helsinki and is reviewed and approved by the ISS Ethics committee and by the Ethics committees of each reference centres involved in the project.

### Registry governance

The Italian PWS Registry is administered by a dedicated SC whose member are selected among researchers from ISS (experts in Registry development and governance), clinicians from the hospitals involved in the care of PWS, and rare disease communities including PWS patient groups, parents, caregivers. The SC main aims are (1) to select inclusion criteria; (2) to validate quality of data within the registry; (3) to analyse patients’ data; (4) to eventually submit proposals to run research projects through the Registry; (5) to publish an annual report deeply describing the information and analyses from data collected. SC has regular meeting to discuss about registry governance and data.

### Patients inclusion criteria

The Italian PWS Registry includes patients of all ages with genetically confirmed PWS diagnosis by DNA methylation-specific testing. Patients with a diagnosis that does not meet the agreed definitions are not accepted in the database and are not included in the analyses. Table 1 summarizes PWS collected types (and relative Orphacode).

**Table 1:** PWS collected genotypes and relative ORPHA codes

PWS genotype	# ORPHA code
By means of chr 15q11q13 paternal deletion	98793
By means of chr 15q11q13, *type 1* paternal deletion	177901
By means of chr 15q11q13, *type2* paternal deletion	177904
By means of chr 15 uniparental maternal disomy	98754
By means of a imprinting center mutation	177910
By means of a point mutation	398069
By means of a chromosomal translocation	177907

### Data anonymization and quality control (QC)

Data form patients are anonymous and each patient is included within the registry through the use of a specific code. Registry administrators continually review data. On an ongoing basis, data points within the registry are curated for date of birth, and diagnosis. Any errors that are identified are corrected by Registry administrators, after a direct discussion with the person who manages the account. Additionally, automatic internal QC have been included and developed within the registry web based platform and are totally compliant with current recommendation [11].

### Data set

It includes mandatory and optional fields within the following variables: demographics; diagnosis and genetics; patient status; therapy; quality of life (QoL); mortality.

### Participating centres

The clinicians of all Italian PWS reference centres were invited to participate. Agreements for data transfer, processing and protection were requested and obtained. Patients data are included only after full written informed consent is obtained; data reflect routine clinical practice and does not require any additional investigations to be carried out over and above standard care. Centres involved in 2019–20 patient’s enrolment are listed in Table 2.

**Table 2:** PWS Italian Centres (N = 10) and relative patient’s distribution

Name of the Italian Referral center per Region	N of patients	%
AOU Policlinico Giovanni XXIII—Bari	3	1.8
AOU Federico II—Napoli	13	7.9
IRCCS Associazione Oasi Maria SS—Troina (EN)	14	8.5
IRCCS Burlo Garofolo—Trieste	4	2.4
IRCCS Istituto Auxologico Italiano—Piancavallo (VB)	64	38.8
IRCCS Ospedale Pediatrico Bambino Gesù—Roma	2	1.2
IRCCS Ospedale San Raffaele—Milano	14	8.5
IRCCS Ospedale Pediatrico IRCCS G. Gaslini—Genova	14	8.5
Ospedale Regina Margherita—AOU Città della Salute—Torino	20	12.1
Policlinico S.Orsola-Malpighi—Bologna	17	10.3
Total of patients	165	100

## Results

### Demography, diagnosis, and genetics

A total of 165 patients were enrolled into the Italian PWS registry in 2019–2020 period: 50.3% (N = 83) of patients was female and 49.7% (N = 82) male. In Italy it has been estimated a total number of 2000 patients with PWS. Our registry currently has an estimated coverage of 8.25%.

Patients were collected by 10 different Italian PWS referral centres (Table 2); these Centres collect the major part of PWS patients in Italy. In 2021, 4 additional Centres (from Sicily, Tuscany, Lombardy and Lazio regions) actively participated to Italian PWS registry activities but data from their centres are not included in the present paper.

Seventy-five subjects were 17 years of age or less, while 90 were adults. Interestingly, first PWS diagnosis (by genetic analyses) was provided in 50 different centres, which are not always the same in which the patients are currently followed-up (10 out of 50).

**Fig. 1:**
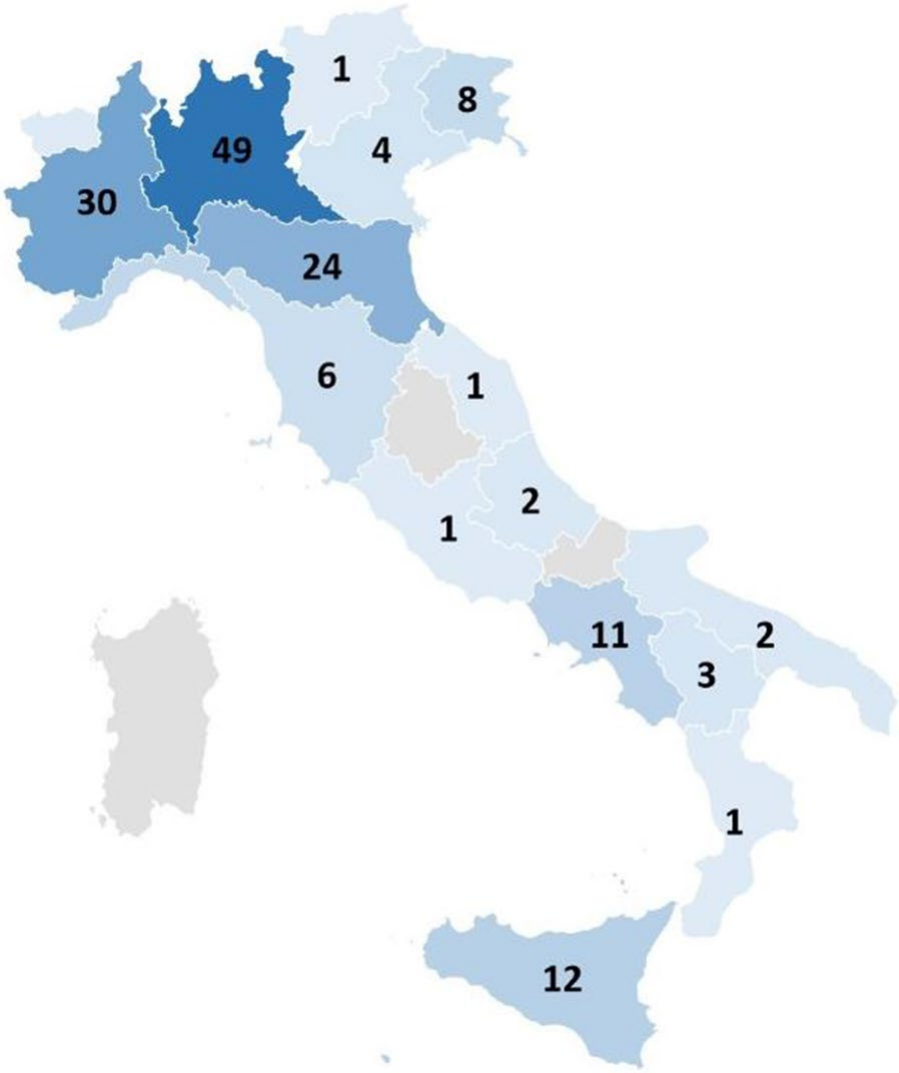
PWS patient’s geographical distribution according to the Italian Region of residence

Age at genetic diagnosis ranged from 0 to 45 years; average was 4.6 years (median 0.9 years). Table 3 describes number of PWS diagnoses by means of molecular genetic testing performed by Methylation-specific multiplex ligation-dependent probe amplification (MSMLPA) or DNA polymorphism study by microsatellite analysis. Ninety-four subjects had interstitial deletion of the proximal long arm of paternal chromosome 15 (del15q11.2-q13), 56 had uniparental maternal disomy for chromosome 15, three presented an imprinting centre defect and one had a de novo translocation involving chromosome 15. In addition, a positive methylation test was demonstrated in the remaining 11 individuals but the underlying genetic defect was not identified. Figure 1 shows patient’s geographical distribution according to the Italian Region of residence.

**Table 3:** Number of patients with a PWS diagnosis by means of molecular genetic testing performed

Genetic testing performed	N of patients	%
PWS (by mean of a positive methylation test)	11	6.7
PWS (by mean of 15q11q13 paternal deletion)	94	57.0
PWS (by mean of maternal uniparental disomy, UPD 15 chromosome)	56	33.9
PWS (by mean of the imprinting center defect)	2	1.2
PWS (by mean of a point mutation)	1	0.6
PWS (by mean of a chromosomal translocation)	1	0.6
Total	165	100

### Clinical data

Our preliminary data show that 105 subjects out of 165 (63.6%) were characterized by compulsive food-seeking and hyperphagia, with a higher percentage in adults (87.8%) than in subjects under the age of 18 (34.7%). As a result, 90 patients (54.5%) developed morbid obesity [18, 19], including 24 children and adolescents (32.0%) and 66 adults (73.3%). Table 6 shows number and percentage of patients with an altered glucose metabolism (left side, 32.3%). Impaired fasting glucose (IFG), impaired glucose tolerance (IGT), diabetes mellitus type 1 (DMT1) and type 2 (DMT2) percentages are also indicated. Most cases with altered glucose metabolism involved adult patients, with the exception of 4 children and adolescents with IGT. Thirty-two patients with altered glucose metabolism were undergoing pharmacological therapy, while the remaining 22 subjects were on diet only. Overall, 40% of patients had an altered lipid profile, single or combined (Table 4, right side), 75.6% being adults and 24.4% children and adolescents. Eight subjects were treated with statins, one with fenofibrate and one with polyunsaturated fatty acids.

**Table 4:** Patients (N and %) with altered glucose [17] and lipid metabolism

	Patients with altered glucose and lipid metabolism			
	Altered glucose metabolism		Altered lipid metabolism	
	n	%	n	%
Missing data	4	2.4	5	3.0
No	103	62.4	93	56.4
Unknown	3	1.8	1	0.6
Yes	55	33.3	66	40.0
	*IFG 4/55*	7.3	*Hypercholesterolemia 42/66*	63.6
	*IGT 24/55*	43.6	*Hypertriglyceridemia 10/66*	15.2
	*DMT1 0/55*	0.0	*Mixed hyperlipidemia 5/66*	3.0
	*DMT2 27/55*	49.1	*HDL hypocholesterolemia 23/66*	13.9
Total	165	100.0	165	100.0

Concerning endocrine diseases, central hypothyroidism was reported in 20% of patients, while central adrenal insufficiency was present in 10.3% of subjects. Sixty-eight adults had an evaluation of the growth hormone/insulin growth factor (GH/IGF) axis, and 37 of them (54.4%) had GH deficiency (GHD). Overall, 94.7% of children and adolescents and 13.3% of adult patients (59,5% of subjects with GHD) were undergoing GH treatment, respectively (Table 5).

**Table 5:** Number of Patients undergoing GH treatment

	**Patients undergoing GH treatment**			
	**Paediatric age**		**Adult age**	
	**N**	**%**	**N**	**%**
No	3	4.0	54	32.7
Yes	71	94.7	22	13.3
Missing data	1	1.3	14	8.5
Total	75	100	90	55

Finally, 77.6% of patients had typical *psychiatric and behavioural features* of people with PWS. In particular, self-injury was reported in 49.1% of them, while 9.7% of patients were affected by psychiatric disorders, including stereotyped behaviour, obsessive–compulsive disorder, schizophrenia spectrum, etc. (Table 6). All the patients (out of the 165 actually included in the registry) were living at the time of publication.

**Table 6:** Number of patients with behavioural and psychiatric disorders

	Patients with behavioural and psychiatric disorders
	N
Undetermined	3
Sterotyped behavior	1
Obsessive–compulsive disorder	4
Disruptive disorder	1
Impulse-control disorder	1
Other psychotic disorder and intermittent explosive disorder	1
Schizoaffective disorder	1
Suicidal ideation and depressed mood, compulsive food-seeking	1
Obsessive–compulsive disorder, aggressiveness	1
Other psychotic disorder	1
Intermittent explosive disorder and aggressiveness	1
Total	16

## Discussion

Advances in e-technologies offer new opportunities to collect, integrate and share data and information from different sources and to advance the understanding of diseases thus eventually supporting the development of diagnostic tools as well as new treatments.

In the last years many papers have been published on the topic “rare disease registry”: a recent search (August 2022) on PubMed using “rare diseases” AND “registry” shows a 100% increment of publications in 2016–2021 period (https://pubmed.ncbi.nlm.nih.gov/?term=rare+disease+AND+registry).

Setting up a registry includes a tremendous variability in scope, size, and resource requirements for registries. This may require the collection of limited or extensive amounts of data, being active for short or long periods of time. Most importantly, the scope and focus of a registry may be well defined and, at the same time, adapted over eventual new research questions. Finally, registries require good planning to be successful [20].

The Italian PWS registry was established in 2019 following an official request by the Italian Patients Federation PWS-Onlus and by the scientific PWS community to better describe the natural history of PWS, to determine clinical effectiveness of health care services and treatments, to monitor the quality of patient care. The final aim of the registry is to improve benefits to patients and their caregivers and to increase scientific knowledge by focusing on: treatment effectiveness, provision of information on the natural history of disease, active monitoring of risk groups and provision of risk stratification, assessment of disease burden, health care planning and identification of areas in health care services policies that require intervention.

In this respect, PWS registry was developed within the frame of a dedicated web-based platform named “RegistRare” established by the National Centre for Rare Diseases at the Istituto Superiore di Sanità (Rome).

Preliminary results are focused on a total of 165 patients enrolled in 2019 – 2020 period (estimated Italian PWS coverage of 8.25%); despite the small number of patients currently included in the analyses of the present paper, our aim is to highlight the registry initiative (the first in Italy for this syndrome) and to compare our preliminary results with the actual scenario.

As expected, our data show that the majority of PWS patients were morbid obese, affecting 54.5% of the individuals. However, it should be noted that this percentage is lower than that previously observed in the Italian population with PWS, both in paediatric and in adult subjects [21]. The possible explanation for these promising results could be the increasing precocity of diagnosis associated with multidisciplinary care at all ages and adequate transition management, achieved in the last decade [22, 23].

If compared with literature [24], our data suggest a higher prevalence of altered glucose metabolism, prevalently in adult subjects. Overall, 40% of patients included in PWS registry had an altered lipid profile, isolated or combined, thus confirming our previous findings in children [25] and in adult patients [26]. Furthermore, data from Italian PWS registry, show that both the alterations of glucose metabolism and those of the lipid profile are mainly treated with diet only, while the propensity to use drugs is low.

Concerning endocrine diseases, hypothyroidism was reported in 20% of patients, similarly to what observed by other Authors [27, 28]. On the other hand, the percentage of patients with adrenal insufficiency was significantly higher than that reported in the literature [29, 30]. This difference may be due to the origin of our results, as they derive from a real-life experience and not from standardized research protocols. However, with the data available from the present study, we are actually unable to give definitive answers to this discrepancy.

The great majority of children and adolescents with PWS was undergoing GH therapy, according to the international consensus guidelines [31]. As for adult subjects, only 13.3% of them (59.5% of subjects with GHD) are treated with GH. In this context, previous studies have shown that GH administration exerts beneficial effects both in PWS adults with and without GHD [32, 33]. However, few data are currently available on the natural history of adults with PWS with or without GH therapy. From this point of view, we strongly believe that data from a well-defined registry could be extremely useful in better understand the potential benefits of GH treatment in these subjects.

Italian PWS registry also collects mortality and its causes data; all the patients (out of 165 actually included in the registry) were living at the time of publication. It will be extremely important and interesting to include in the future data regarding the average life expectancy of people living with PWS and, eventually, to underline its variation from country to country. Such knowledge might inform the effectiveness of current diagnosis and treatment.

Finally, our results showed that behavioural and psychiatric disorders affect more than three-quarters of our population with PWS [34]. These data confirm that managing the behavioural and psychiatric phenotype represents a daily challenge for the care of these patients, for both healthcare professionals and caregivers. Also in this case the registry allows to focus on the area of intervention in which the utmost surveillance and the greatest commitment of resources are required.

## Conclusion

Despite the important difficulties in the planning phase of a registry setting up (i.e. strategies for patient’s enrolment and retention, limited resources and voluntary rather than mandatory participation, etc.), use of this tool is extremely important for collecting clinical and epidemiologic data, assessing the clinical management including the diagnostic delay, improving patients’ care and fostering research to identify new therapeutic solutions.

The Italian PWS registry provides the flexibility of both updatable as well as longitudinal surveys. This reflects on the possibility to capture the natural history of onset and severity of PWS symptoms and their treatment throughout a participant’s life, as well as to track changes over shorter periods of time for aspects, of the PWS phenotype such as obesity and its comorbidities, endocrine alterations, behavioural aspects or psychiatric disorders. With the ongoing increasing number of participating centres (and consequently of included patients) within the registry, it will be interesting to monitor and update these data year by year.

With growing participation, the Italian PWS Registry aims at strengthening collaborations with national and international researchers, clinicians, patients and other partners to advance the understanding of PWS, direct efforts in basic and clinical research, and support the development of novel therapies for this challenging disorder.

Another future direction for PWS registry is represented by the aim of increasing patient involvement even in the data collection process; our final aim would be to improve their perception on medical registries and data collection, by the addition of variables that matter to the registered individuals. Also, for certain domains, like patient care, subjects involvement can be translated into a gain in the capacity to collect support care data in real time [35] and favouring medical registries in the delicate balance between the right to privacy and the need for information in the public health domain.

## Data Availability

Data sharing not applicable to this article as no datasets were generated or analysed during the current study.

## Abbreviations

PWS: Prader–Willi syndrome
NCRD: National Centre for Rare Diseases
ISS: Istituto Superiore di Sanità
GDPR: European Union General Data Protection Regulation
FAIR: Findable, accessible, interoperable, and reusable
SC: Scientific committee
IC: Informed consent
QC: Quality control
QoL: Quality of life
MSMLPA: Methylation-specific multiplex ligation-dependent probe amplification
IFG: Impaired fasting glucose
IGT: Impaired glucose tolerance
DMT1: Diabetes mellitus type 1
DMT2: Diabetes mellitus type 2
GH/IGF: Growth hormone/insulin growth factor

## References

[1] Angulo MA, Butler MG, Cataletto ME (2015). Prader–Willi syndrome: a review of clinical, genetic, and endocrine findings. J Endocrinol Investig.

[2] Cassidy SB, Schwartz S, Miller J, Driscoll DJ (2012). Prader–Willi syndrome. Genet Med.

[3] Godler DE, Butler MG (2021). Special issue: genetics of Prader–Willi syndrome. Genes.

[4] Bar C, Diene G, Molinas C, Bieth E, Casper C, Tauber M (2017). Early diagnosis and care is achieved but should be improved in infants with Prader–Willi syndrome. Orphanet J Rare Dis.

[5] Butler MG, Hartin SN, Hossain WA, Manzardo AM, Kimonis V, Dykens E, Gold JA, Kim SJ, Weisensel N, Tamura R (2019). Molecular genetic classification in Prader–Willi syndrome: a multisite cohort study. J Med Genet.

[6] Pacoricona Alfaro DL, Lemoine P, Ehlinger V, Molinas C, Diene G, Valette M, Pinto G, Coupaye M, Poitou-Bernert C, Thuilleaux D, Arnaud C, Tauber M (2019). Causes of death in Prader–Willi syndrome: lessons from 11 years' experience of a national reference center. Orphanet J Rare Dis.

[7] Driscoll DJ, Miller JL, Schwartz S, Cassidy SB. Prader–Willi Syndrome. In: Adam MP, Ardinger HH, Pagon RA, Wallace SE, Bean LJH, Gripp KW, Mirzaa GM, Amemiya A, editors. GeneReviews® [Internet]. University of Washington, Seattle; Seattle (WA): Oct 6, 1998; Passone CBG, Pasqualucci PL, Franco RR, Ito SS, Mattar LBF, Koiffmann CP, Soster LA, Carneiro JDA, Cabral Menezes-Filho H, Damiani D. Prader–Willi syndrome: what is the general pediatrician supposed to do? - a review. Rev Paul Pediatr. 2018;36(3):345–352. 10.1590/1984-0462/;2018;36;3;00003 PMC6202899 30365815

[8] Bohonowych J, Miller J, McCandless SE, Strong TV (2019). The global Prader–Willi syndrome registry: development, launch, and early demographics. Genes.

[9] Aypar U, Hoppman NL, Thorland EC, Dawson DB (2016). Patients with mosaic methylation patterns of the Prader–Willi/Angelman Syndrome critical region exhibit AS-like phenotypes with some PWS features. Mol Cytogenet.

[10] Heksch R, Kamboj M, Anglin K, Obrynba K (2017). Review of Prader–Willi syndrome: the endocrine approach. Transl Pediatr.

[11] Kodra Y, Minelli G, Rocchetti A, Manno V, Carinci A, Conti S, Taruscio D, National Rare Diseases Registry Collaborating Group (2019). The Italian National Rare Diseases Registry: a model of comparison and integration with Hospital Discharge Data. J Public Health.

[12] Communication from the commission to the European parliament, the council, the European economic and social committee and the committee of the regions on Rare Diseases: Europe's challenges. https://ec.europa.eu/health/ph_threats/non_com/docs/rare_com_en.pdf. Accessed 6 Sept 2022.

[13] Taruscio D, Mollo E, Gainotti S, Posada de la Paz M, Bianchi F, Vittozzi L (2014). The EPIRARE proposal of a set of indicators and common data elements for the European platform for rare disease registration. Arch Public Health.

[14] Kölker S, Gleich F, Mütze U, Opladen T (2022). Rare disease registries are key to evidence-based personalized medicine: highlighting the European experience. Front Endocrinol (Lausanne).

[15] Italian Prader Willi syndrome registry platform. www.registrare.org/schede-11-registro_prader_willi. Accessed 6 Sept 2022.

[16] European Platform on Rare Disease Registration (EU RD Platform). https://eu-rd-platform.jrc.ec.europa.eu/it. Accessed 6 Sept 2022.

[17] Wilkinson M, Dumontier M, Aalbersberg I (2016). The FAIR Guiding Principles for scientific data management and stewardship. Sci Data.

[18] Graling P, Elariny H. Perioperative care of the patient with morbid obesity. AORN J. 2003;77(4):802–5; 808–19; quiz 820–1, 823–4. 10.1016/s0001-2092(06)60799-0 12705735

[19] Alberti KGMM, Zimmet P, Shaw J (2006). Metabolic syndrome—a new world-wide definition. A consensus statement from the international diabetes federation. Diabet Med.

[20] Gliklich RE, Dreyer NA, Leavy MB, editors. Registries for evaluating patient outcomes: a user's guide [Internet]. 3rd ed. Rockville (MD): Agency for Healthcare Research and Quality (US); 2014 Apr. Report No.: 13(14)-EHC111. 24945055

[21] Grugni G, Crinò A, Bosio L, Corrias A, Cuttini M, De Toni T, Di Battista E, Franzese A, Gargantini L, Greggio N, Iughetti L, Livieri C, Naselli A, Pagano C, Pozzan G, Ragusa L, Salvatoni A, Trifirò G, Beccaria L, Bellizzi M, Bellone J, Brunani A, Cappa M, Caselli G, Cerioni V, Delvecchio M, Giardino D, Iannì F, Memo L, Pilotta A, Pomara C, Radetti G, Sacco M, Sanzari A, Sartorio A, Tonini G, Vettor R, Zaglia F, Chiumello G; Genetic Obesity Study Group of Italian Society of Pediatric Endocrinology and Diabetology (ISPED). The Italian National Survey for Prader–Willi syndrome: an epidemiologic study. Am J Med Genet A. 2008;146A(7):861–72. 10.1002/ajmg.a.32133 18203198

[22] Kimonis VE, Tamura R, Gold JA, Patel N, Surampalli A, Manazir J, Miller JL, Roof E, Dykens E, Butler MG, Driscoll DJ (2019). Early diagnosis in Prader–Willi syndrome reduces obesity and associated co-morbidities. Genes (Basel).

[23] Paepegaey AC, Coupaye M, Jaziri A, Ménesguen F, Dubern B, Polak M, Oppert JM, Tauber M, Pinto G, Poitou C (2018). Impact of transitional care on endocrine and anthropometric parameters in Prader–Willi syndrome. Endocr Connect.

[24] Fintini D, Grugni G, Bocchini S, Brufani C, Di Candia S, Corrias A, Delvecchio M, Salvatoni A, Ragusa L, Greggio N, Franzese A, Scarano E, Trifirò G, Mazzanti L, Chiumello G, Cappa M, Crinò A; Genetic Obesity Study Group of the Italian Society of Pediatric Endocrinology and Diabetology (ISPED). Disorders of glucose metabolism in Prader–Willi syndrome: results of a multicenter Italian cohort study. Nutr Metab Cardiovasc Dis. 2016;26(9):842–7. 10.1016/j.numecd.2016.05.010 27381990

[25] Brambilla P, Crinò A, Bedogni G, Bosio L, Cappa M, Corrias A, Delvecchio M, Di Candia S, Gargantini L, Grechi E, Iughetti L, Mussa A, Ragusa L, Sacco M, Salvatoni A, Chiumello G, Grugni G; Genetic Obesity Study Group of the Italian Society of Pediatric Endocrinology and Diabetology (ISPED). Metabolic syndrome in children with Prader–Willi syndrome: the effect of obesity. Nutr Metab Cardiovasc Dis. 2011;21(4):269–76. 10.1016/j.numecd.2009.10.004 20089384

[26] Grugni G, Crinò A, Bedogni G, Cappa M, Sartorio A, Corrias A, Di Candia S, Gargantini L, Iughetti L, Pagano C, Ragusa L, Salvatoni A, Spera S, Vettor R, Chiumello G, Brambilla P (2013). Metabolic syndrome in adult patients with Prader–Willi syndrome. Nutr Metab Cardiovasc Dis.

[27] Iughetti L, Vivi G, Balsamo A, Corrias A, Crinò A, Delvecchio M, Gargantini L, Greggio NA, Grugni G, Hladnik U, Pilotta A, Ragusa L, Salvatoni A, Wasniewska M, Weber G, Predieri B (2019). Thyroid function in patients with Prader–Willi syndrome: an Italian multicenter study of 339 patients. J Pediatr Endocrinol Metab.

[28] Pellikaan K, Snijders F, Rosenberg AGW, Davidse K, van den Berg SAA, Visser WE, van der Lely AJ, de Graaff LCG (2021). Thyroid function in adults with Prader–Willi syndrome; a cohort study and literature review. J Clin Med.

[29] Corrias A, Grugni G, Crinò A, Di Candia S, Chiabotto P, Cogliardi A, Chiumello G, De Medici C, Spera S, Gargantini L, Iughetti L, Luce A, Mariani B, Ragusa L, Salvatoni A, Andrulli S, Mussa A, Beccaria L, Study Group for Genetic Obesity of Italian Society of Pediatric Endocrinology and Diabetology (SIEDP/ISPED) (2012). Assessment of central adrenal insufficiency in children and adolescents with Prader–Willi syndrome. Clin Endocrinol (Oxf).

[30] Rosenberg AGW, Pellikaan K, Poitou C, Goldstone AP, Høybye C, Markovic T, Grugni G, Crinò A, Caixàs A, Coupaye M, Van Den Berg SAA, Van Der Lely AJ, De Graaff LCG (2020). Central adrenal insufficiency is rare in adults with Prader–Willi syndrome. J Clin Endocrinol Metab.

[31] Deal CL, Tony M, Höybye C, Allen DB, Tauber M, Christiansen JS, 2011 Growth Hormone in Prader–Willi Syndrome Clinical Care Guidelines Workshop Participants (2013). Growth Hormone Research Society workshop summary: consensus guidelines for recombinant human growth hormone therapy in Prader–Willi syndrome. J Clin Endocrinol Metab.

[32] Grugni G, Sartorio A, Crinò A (2016). Growth hormone therapy for Prader–willi syndrome: challenges and solutions. Ther Clin Risk Manag.

[33] Höybye C, Holland AJ, Driscoll DJ, Clinical and Scientific Advisory Board of the International Prader–Willi Syndrome Organisation (2021). Time for a general approval of growth hormone treatment in adults with Prader–Willi syndrome. Orphanet J Rare Dis.

[34] Schwartz L, Caixàs A, Dimitropoulos A, Dykens E, Duis J, Einfeld S, Gallagher L, Holland A, Rice L, Roof E, Salehi P, Strong T, Taylor B, Woodcock K (2021). Behavioral features in Prader–Willi syndrome (PWS): consensus paper from the International PWS Clinical Trial Consortium. J Neurodev Disord.

[35] Nelson EC, Dixon-Woods M, Batalden PB, Homa K, Van Citters AD, Morgan TS (2016). Patient focused registries can improve health, care, and science. BMJ.

